# A New Method of Inviting Sleep

**Published:** 1897-10

**Authors:** J. B. Learned

**Affiliations:** Northampton, Mass.


### A NEW METHOD OF INVITING SLEEP.

By J. B. LEARNED, M. D., NORTHAMPTON, MASS.

A violent collision with the frozen earth, the result of my first drive with a newly purchased horse in 1880, took me out of the busy life of the every day and night practitioners. For many years, instead of experimenting upon a willing and paying class of the laity, I was experimenting upon an unwilling and non-paying member of the profession. I did not sample all the remedies of the "Dispensatory," but I sampled many remedies outside the "Dispensatory." Hot water, cold water, inside and out; lack of food and surplus of food; gymnastics in my room and gymnastics with the wood saw in the basement in the night time, brisk walking in the halls and around the square before retiring, friction direct and indirect, long deep inspirations with and without the numberless mental occupations, as varied as the physical; all these I tried faithfully. I also read about insomnia, its causes and remedies. It was during this blank interval, while I waited for power to return to me, that I raised the inquiry: Can we devise any means to turn off the belts from this little fragment of brain that insists on its automatic excursions day and night—this perpetual motion of a few cells of gray matter, that obstructs rest and prevents repair of the great whole? Can we by counteraction set up a motion elsewhere after retiring, that will bring an equilibrium of arterial and vital current so that sleep will come to our relief?

During this frame of mind, I experimented and practiced with muscle and will in many and divers ways after retiring. I had the whole bed, length and breadth. I directed various contractions and relaxations, and finally reached the conclussion that a systematized and well-ordered method of muscular and mental activity would soon bring the conditions required—a sense of fatigue that precedes and invites sleep. A recumbent position furnished the best opportunity. Once asleep the point is gained. Who has not been dull and almost asleep before retiring, but wide awake immediately after disrobing and experiencing the gentle shock of the fresh sheets and changed posture?

Is it necessary to recite here the advantages of proper conditions of atmosphere and temperature of the sleeping-room? I will assume not, but will say that open windows at all seasons, heat never turned on in my sleeping-room and moderate bed-covering has come to be a necessity with me. This is my method:

Lying upon my back, with or without pillow, I reach for the foot-board and head-board at the same time. This brings into use many muscles that have not been on active duty during the day. I now raise the head half an inch, enough to realize that it has more weight than I first supposed. At the same time, by will power, I direct the respiratory process to a slower and deeper movement. I order about eight inspirations deep and full, in place of sixteen per minute. Every inspiration is recorded, counted. Thus the process begins of inviting the forces into new channels and relieving the old. At the expiration of ten to twenty inspirations the head has become so heavy you want to drop it. This you do. Immediately the right foot is raised a half inch from its resting place. The reach for the foot-board and head-board continues; the count of slow, deep inspirations continues; the sense of fatigue of muscles engaged in lifting the foot and holding up the coverings continues. Here, as before, the foot, like the head, has become a dead weight and must go down. Now, immediately the left foot is elevated with all the previous conditions remaining. The reach downward and upward of foot and head is kept up, so far as power will permit without exhaustion. This foot remains up for the same length of time, the respirations being the clock work. It goes down. You may now relieve the reach for foot and head boards and use the muscles to elevate the trunk, holding it by resting upon heels and head and shoulders. This elevation of the central part of the body and rest upon the two extremes will call for change, as all the former positions have done. By the same clock you have the time marked off and the body is again flat upon the back waiting new orders. Turn now to the right side, reaching for the head and foot boards as before, and elevate the head half an inch by use of the lateral muscles of the neck and chest. At the expiration of the time the head goes down and the foot goes up by use of the lateral muscles. Change now to the left side, and repeat what has just been accomplised by the muscles of the opposite side. You have now assumed eight positions and used a large majority of the whole number of muscles in carrying out your dictations. If you have not fallen asleep before this cycle is completed you may begin again and go over the same round. If you have already gone to sleep you will not be required to.

Other methods of procedures would answer the same purpose, undoubtedly, with the respirations guarded and uniformity observed, mind and muscle constantly occupied. There should be no periods of rest, no vacations. Thus fatigue comes inevitably and sleep follows. I know of no means so ready, so much at command, any time and anywhere, so inexpensive and so absolutely certain to induce sleep as this routine of mental and muscular exercise. It involves a principle. Following it, sleep appears to be inevitable. There is but one drawback; it requires some exertion, continuous mental and muscular exertion. The indolent will find it unattractive.

Some conditions of heart or nerve center may altogether contra-indicate this method. The length of time employed in the several positions will vary according to the make-up of the individual. No one rule can apply to the robust and the exhausted as to the time spent in a given exercise, only that which measures the power of endurance. It is the sense of general weariness following persistent effort that brings the desired result.—*Journal American Medical Association.*

